# Sedation and anesthesia for imaging of the infant and neonate—a brief review

**DOI:** 10.1007/s00247-024-05995-5

**Published:** 2024-07-26

**Authors:** Forrest P. Beaulieu, Gabriel Zuckerberg, Kristen Coletti, Emily Mapelli, John Flibotte, Spoorthi Sampath, Misun Hwang, Elizabeth T. Drum

**Affiliations:** 1 Department of Anesthesiology and Critical Care, Perelman School of Medicine, University of Pennsylvania, Philadelphia, PA 19104 USA; 2 Division of Neonatology, Department of Pediatrics, Children’s Hospital of Philadelphia, Philadelphia, PA 19104 USA; 3 Department of Pediatrics, Children’s Hospital of Philadelphia, Philadelphia, PA 19104 USA; 4 Department of Radiology, Children’s Hospital of Philadelphia, University of Pennsylvania, Philadelphia, PA 19104 USA; 5 Children’s Hospital of Philadelphia, Philadelphia, PA 19104 USA

**Keywords:** Anesthesia, Infant, Neonatology, Newborn, Radiology

## Abstract

Sedation and anesthesia are often required in order to facilitate collection of high-quality imaging studies free of significant motion artifact for infants and neonates. Provision of safe sedation and anesthesia requires good communication between the ordering provider, radiologist, and anesthesiologist, careful pre-procedural evaluation of the patient, and availability of appropriate and sufficient equipment, drugs, personnel, and facilities. There are many additional factors to be considered for provision of safe sedation or anesthesia for infants and neonates—it is ideal to involve a fellowship-trained pediatric anesthesiologist in the planning and carry-out of these plans. In this review, we discuss some of the basic definitions of sedation and anesthesia, requirements for safe sedation and anesthesia, and many of the germane risks and additional considerations that factor into the delivery of a safe sedation or anesthesia plan for the imaging of an infant or neonate.

**Supplementary Information:**

The online version contains supplementary material available at 10.1007/s00247-024-05995-5.

## Introduction

The objective of sedation and anesthesia as it pertains to pediatric imaging is simple—to facilitate an optimal image or study to benefit the patient while maintaining the safety and comfort of the child [1–4]. While simply stated, accomplishing this goal is more complex: the American Academy of Pediatrics (AAP) defines the requirements for safe sedation of children for diagnostic and therapeutic procedures as requiring a systematic approach that includes medical evaluation, appropriate fasting and preparation, sufficient personnel, equipment, and facility resources, and a trained and knowledgeable medical provider [3]. Need for sedation and general anesthesia for infant and neonatal imaging is increasing as the availability of high-level diagnostic imaging technologies such as computed tomography (CT), magnetic resonance imaging (MRI), nuclear medicine, interventional radiology studies, and other high-resolution techniques expands [5]. Performing high-quality imaging free of motion artifact is particularly difficult in many children without the aid of sedation. Safe administration of sedation and anesthesia to infants and neonates requires special consideration. Their small size makes airway management and other procedures more technically challenging; they metabolize medications differently and have differences in cardiopulmonary physiology when compared to older children or adults.

Protocols for sedation and anesthesia for imaging of the infant vary across institutions [6–9]. Although several organizations such as the AAP and the Society for Pediatric Anesthesia have published guidelines and positions on the provision of pediatric anesthesia and sedation care, there is much flexibility in the approach to this type of care between providers and throughout hospital systems [3, 10].

Provision of sedation or general anesthesia is not without risk. The United States Food and Drug administration (FDA) has issued a warning regarding exposure to anesthesia in children under the age of 3, citing a concern that medications used for sedation and anesthesia may negatively affect the developing brain [11]. It is imperative that radiologists and ordering providers understand the risks of sedation and anesthesia and weigh them against the potential benefits of the intended imaging study so that they may ensure that requested sedated studies are necessary and appropriate for each patient.

## Depth of sedation/anesthesia [1, 12, 13]

Sedation and anesthesia are often conceptualized as a continuum representing successively “deeper” states of depression of consciousness. There is a spectrum of varying patient response to anesthetic medications, and providers must be prepared to care for patients at deeper levels of sedation regardless of what is planned.

### Minimal sedation

Commonly referred to as “anxiolysis,” minimal sedation is a drug-assisted state in which patients maintain normal response to verbal stimulus. No airway or cardiovascular intervention is required. Cognitive and physical coordination may be impaired [1, 13].

### Moderate sedation

Sometimes colloquially referred to as “conscious sedation,” moderate sedation is a drug-induced state in which patients are sleepy but arousable. Patients in this state respond purposefully to non-painful stimuli, including light touch and direct verbal instruction. Patients breathe spontaneously without airway intervention. No cardiovascular intervention is typically required [1].

### Deep sedation

Deep sedation is a pharmacologically induced state in which patients cannot be aroused through gentle stimulus, but may be aroused through painful or repeated stimuli. The ability to maintain airway tone or reflexes, or spontaneously ventilate, may be impaired and require intervention. Cardiovascular function is typically intact [14].

### General anesthesia

General anesthesia is a pharmacologically induced state of unconsciousness during which patients do not rouse to even painful stimulus. It is typical for patients to require invasive airway support and mechanical intubation. Hemodynamics and cardiovascular function often require some level of pharmacologic support. Patients are often akinetic (unmoving), either through pharmacologic suppression of response to stimulus or through direct neuromuscular blockade [14, 15].

## Medication considerations and risks of sedation and anesthesia

A definition of anesthesia is a drug-induced, reversible state of unconsciousness, unawareness, analgesia, and akinesis [16]. The practice of anesthesia generally involves the consideration and use of medication to ensure patient safety and comfort during their medically necessary procedure. Adequate patient participation in radiology studies typically involves some degree of cooperation and immobility. Many adults and older children are able to comply with these needs with no or minimal pharmacologic assistance, but neonates and infants often cannot, unless the patient and study are amenable to the “feed-and-swaddle” technique to achieve relative immobility. In addition to administration of medications, continuous monitoring of the patient is an essential component of anesthesia care.

Medications used by anesthesiologists aim to provide amnesia, analgesia, and akinesis. Amnesia can be achieved through the use of medications administered for the express purpose of preventing awareness and recollection of interventions. Commonly used anesthetics may include propofol, ketamine, dexmedetomidine, sevoflurane, isoflurane, and nitrous oxide [17, 18]. Analgesics, used to decrease the patient’s sensation of and response to painful or uncomfortable stimuli, may include opioid drugs such as fentanyl in bolus or infusion form or morphine, nonsteroidal anti-inflammatory drugs (NSAIDs), acetaminophen, or ketamine [19]. Local anesthetics are often used to provide analgesia for painful procedures either by direct infiltration or by customized placement of nerve blocks [20]. Independent of their analgesic effects, these agents often work in synergistic fashion with other sedative agents to augment the patient’s overall anesthetic. It should be noted that most diagnostic radiologic procedures are not painful, and analgesia may not be required. Akinesis, or immobility, can be achieved through multiple modalities; medications for sedation and analgesia are often sufficient to prevent patient movement that would interfere with radiology studies, but anesthesiologists may choose to ensure complete immobility through use of paralytic agents like rocuronium or vecuronium [21]. Note that even with general anesthesia and paralysis, there will be respiratory and cardiac motion. In addition, there is often a need for respiratory support for patients under deep sedation or general anesthesia [22].

There are several key differences in neonatal and infant physiology that affect how medications for anesthesia and sedation must be dosed. Neonates have a comparatively high metabolic rate and high minute alveolar ventilation—this leads to increased uptake of volatile anesthetic agents such as sevoflurane when compared to older patients [21, 23]. The dosing of volatile anesthetics is also dependent on the age and degree of prematurity of the infant [24]. Neonates and infants, in comparison with other children, have decreased protein binding and hepatic and renal function. There are also differences in the distribution of cardiac output to the liver and kidneys of neonates, further affecting drug metabolism and excretion. Neonates (and older infants, to a lesser extent) typically have a longer elimination half-life than older children, and this must be considered when dosing medications for anesthesia [19, 25–27]. Unfortunately, data regarding dosing of anesthetic medications for neonates and infants is generally much more sparse than what data exists for adults.

These medications should be used carefully, as all have potential associated toxicities. Anesthetic and analgesic agents may induce bradycardia, impair vascular tone leading to hypotension, and diminish respiratory drive and pharyngeal tone [28, 29]. Chemical paralysis guarantees that the patient will not be able to spontaneously breathe for a significant amount of time. Therefore, use of these agents demands invasive airways and mechanical ventilation throughout the procedure and until the impact of administered medications has resolved. Intubation and ventilation incur a risk of injury to the patient’s airway and lungs and can interfere significantly with the patient’s hemodynamic status [30, 31]. Intravenous access is almost always required for safe administration of anesthesia—intravenous access can be difficult and may require ultrasound guidance, and infiltration can lead to drug extravasation and tissue injury. Use of vaso- and cardio-active medications requires peripheral and sometimes central vascular access; introduction of these lines poses risks to patients including infection, bleeding, damage to vascular and other local structures, and potential tissue injury. Immobile patients are at risk of neurovascular and pressure injuries related to positioning. Administration of general anesthesia outside of the operating room may incur additional risk [32, 33]. In addition, the developmental impact of single and repeat anesthetic encounters in the immature brain remains an area of significant focus. In short, anesthesia is often necessary to achieve a high-quality radiology study, but is not without risk, which must be considered against the potential benefit of any requested study.

As with any aspect of medical and surgical care, the risks and benefits of sedation or anesthesia must be thoroughly discussed with each patient’s guardians/caregivers, and their consent obtained. Caregiver discussion and consent is a required part of sedation and anesthesia, and procedures should not proceed without it, except in rare circumstances in which the child’s life is at risk and a caregiver is unreachable.

## Evaluating the need for sedation or anesthesia for imaging procedures

A tailored, patient-specific approach is critical when preparing a patient for an imaging procedure. The radiologist, ordering provider, and sedation/anesthesia team must incorporate procedure and patient-specific factors to identify what method, if any, of sedation or anesthesia is appropriate to accomplish the procedure. It is imperative that the diagnostic question is clear and that the proposed imaging will answer the question at hand, especially if sedation or anesthesia is required to obtain the imaging. As all anesthetic medications have systemic effects, a holistic, system-based pre-procedural review of the patient’s physiology and medical comorbidity is mandatory to ensure the delivery of safe care [34]. This pre-procedural evaluation, tailored to the specific procedure that is scheduled, typically includes careful chart review, a targeted collection of medical history with the patient and caregiver, and a physical exam [34].

Procedure-specific factors include length of imaging procedure, location of procedure, tolerance for patient movement, requirement for patient participation, and technical components of the imaging study which may be impacted by equipment or procedures necessary for sedation. When considering these factors, it is clear that many techniques, such as plain film radiography and ultrasound do not require any sedation at all, while others, such as complex MRI sequences and nuclear medicine scans, almost exclusively necessitate some level of sedation or anesthesia, and yet others such as dynamic fluoroscopic exams may or may not require sedation or anesthesia, depending on the exam and the patient’s status or cooperativity. Exams that are brief in nature and require minimal cooperation by the patient are often successful without sedation or anesthesia. Exams that require absolute stillness, are lengthy, and require unusual positioning or locations that may exacerbate claustrophobia (though this specific concern is not germane to the neonate and infant population) are much more likely to require sedation or anesthesia. For studies that do not require breath holding or stillness for greater than a few minutes, patients may be appropriate candidates for “natural airway” sedation, where the patient may remain spontaneously breathing and without an invasive airway device. Radiographic studies, most CT protocols, most ultrasonography and echocardiography studies, and certain MRI studies are typically amenable to these types of minimal to moderate sedation, or often to no sedation at all, depending on the demeanor of the child and the length of the study. While older children may be able to maintain appropriate calmness and stillness through primarily nonpharmacologic measures, most infants require at least minimal sedation to participate effectively in such studies.

For procedures that require total immobility or periods of breath holding to facilitate a quality image, however, general anesthesia with a secured airway and possibly neuromuscular blockade may be required. These include certain MRI studies, CT studies assessing lung parenchyma for metastases or other resolution-sensitive pathology, complex nuclear medicine scans, and interventional radiology procedures where patient movement may be dangerous or make the procedure impossible. A pre-procedure discussion between the radiologist and anesthesiologist is essential to ensure that the optimal sedation or anesthetic course is selected for each imaging study. It is ideal to understand special requests from radiologist such as positioning other than supine and need for breath holds prior to making a final sedation or anesthesia plan. Requests for specific heart rate and blood pressure ranges or specific medications require careful consideration of the patient’s baseline hemodynamic state. In addition, consideration of patient-specific factors is also crucial; variation in age, developmental status, and comorbid medical conditions (such as craniofacial, airway, cardiac, pulmonary, hematologic and oncologic issues, and neurologic disease) may require alterations to the sedation or anesthesia plan for a given imaging modality. For example, children with severe obstructive sleep apnea may require a general anesthetic with endotracheal tube, as opposed to a natural airway sedation that may predispose them to dangerous obstruction and hypoxemia.

The need for sedation or general anesthesia for MRI is relatively common for pediatric patients. As discussed, neonates and infants have unique physiologic concerns that require special expertise. MRI safety concerns, length of study, and the physical barrier between patient and monitoring team increase the level of vigilance needed. Other modalities such as nuclear medicine and interventional radiology also have specific concerns. Most nuclear medicine studies require injection of isotope before the study which requires IV access and careful timing. Interventional radiology procedures are often painful and therefore a higher percentage of patients require sedation or general anesthesia. Access to the patient during many radiology procedures may be limited by safety issues or the radiology equipment itself. Patients with oncologic processes may have acute medical concerns due to active treatment. The principles of sedation and general anesthesia remain the same: maintenance of a stable and unobstructed airway, hemodynamic stability, and pain control. The specific requirements of the study and the location where the study occurs require understanding of the needs by the team providing sedation or general anesthesia.

As has been previously described, sedation and general anesthesia carry both acute and potentially long-term risks. Therefore, determination of the minimal amount necessary to safely and effectively complete the imaging study is prudent. For many patients up to the age of 6 months, a state of calm and akinesis necessary for the success of the study can be achieved simply by taking advantage of the tendency of infants to fall asleep when they are fed and warm, known as “feed-and-swaddle.” This technique has been shown to be broadly effective among infants up to 6 months undergoing brain MRI in both inpatient and outpatient populations [35]. Other non-pharmacological techniques include use of an immobilizer to reduce body movement, use of ear muffs, use of a pacifier with or without a glucose/sucrose mixture, and timing of imaging for when the patient is already asleep [36].

A systematic review and meta-analysis of non-pharmacological strategies to obtain MRI images in infants showed that these techniques are generally exceedingly useful, allowing imaging success in 87% of patients with an average study time of 30 min; however, noted that in cases of medical complexity and need for structural brain imaging, these techniques were less effective [36]. While most of the data regarding these techniques describes MRI studies, it is reasonable to extrapolate to other studies which require prolonged periods of immobility such as nuclear medicine and positron emission tomography (PET) imaging. Therefore, if resources allow, a general approach which attempts non-pharmacologic interventions before the use of sedation or general anesthesia is preferable. Consideration should also be given to splitting up longer studies into discrete segments which can be obtained without sedation or anesthesia.

## Requirements for safe provision of sedation or general anesthesia

When non-pharmacologic interventions fail, or conditions such as swallowing dysfunction, critical illness, or urgent/emergent need for imaging results necessitate the use of sedation or general anesthesia, a holistic, standardized approach is crucial to maximize patient safety. As described previously, administration of sedative/hypnotic agents produce sedative effects on a spectrum which ranges from mild anxiolysis with general preservation of respiratory/cardiovascular function and airway reflexes, to general anesthesia with profound cardiorespiratory depression and blunting of autonomic and airway reflexes. While general dosing strategies to achieve various depths of sedation or anesthesia are available, precise individual responses are unpredictable, and therefore providers need to be prepared to support patients as if they were achieving general anesthesia. To this end, it is critical that providers follow established guidance with regard to NPO (“nil-per-os,” or “nothing by mouth”) time as described by the American Society of Anesthesiologists (ASA) Committee on Standards and Practice Parameters to reduce the risk of pulmonary aspiration of gastric contents (Supplementary Material 1) [37].

Furthermore, assessment of a patient’s underlying medical conditions (commonly assessed using the American Society of Anesthesiologists Physical Status Classification) should be performed, and if possible, measures to optimize a patient before sedation or anesthesia should be undertaken (Supplementary Material 2) [38]. For example, as infants with concurrent or recent viral upper respiratory illness are at increased risk for airway adverse events, consideration should be given to postponement of the study until respiratory symptoms have resolved, if possible [39]. Similarly, in patients with comorbid chronic disease such as chronic lung disease, cardiac disease, and seizure disorders, consultation with subspecialists and consideration for increased post-procedural care and monitoring may be warranted.

In addition to appropriate patient preparation and risk stratification, the presence of required monitoring and rescue equipment, as well as sufficient provider skillset, is absolutely necessary for the safe provision of sedation. Whenever providing deep sedation or general anesthesia, the use of standard monitoring as described by the ASA should be utilized [40]. These include the following:

1. Presence of qualified anesthesia personnel throughout the conduct of all general anesthetics and monitored anesthesia care (sedation)
2. Monitoring of oxygenation (use of oxygen analyzer and pulse oximetry)
3. Monitoring of ventilation (physical examination including chest excursion and auscultation, and qualitative or quantitative capnography)
4. Monitoring of circulation (use of electrocardiogram continuously and measurement of blood pressure and heart rate at least every 5 min)
5. Monitoring of temperature (temperature monitoring when clinically significant changes in body temperature are intended, anticipated, or suspected, or while under general anesthesia for more than 30 min)

As many imaging procedures require the provider to be physically separated from the patient, communication and coordination between the sedation or anesthesia provider, radiologist, and radiology technologist is critical. The use of imaging-compatible monitoring equipment and systems to allow remote monitoring in the control room is required.

The American Academy of Pediatrics (AAP) recommends that the practitioner providing sedation services should be trained in advanced pediatric airway skills necessary to rescue potential complications of sedation, such as provision of continuous positive airway pressure and effective bag mask ventilation [3]. Furthermore, at least one team member should be skilled in vascular access, as many anesthetic courses require intravenous access for provision of anesthesia and/or cardiovascular support. In addition to appropriate practitioner skills, the presence of rescue equipment is mandatory. The AAP recommends the use of a mnemonic memory device, “SOAPME,” to ensure all necessary equipment is available and functional (Supplementary Material 3) [3].

In some institutions, intensive care unit teams may have the expertise and appropriate staffing to provide sedation for patients admitted to their unit. Institutional guidelines usually exist which delineate which patients are appropriately cared for by an ICU team and which patients need to be referred to the anesthesiology department. Examples of need for care by the anesthesiology department may include difficult or critical airway, need for volatile anesthetics, use of vasopressors, special needs beyond the usual scope of care for an ICU patient such as breath holds, potential for rapid or large blood loss, and congenital cardiac disease. In addition, some institutions have specific credentialing requirements for care of neonates and use of certain medications such as propofol, dexmedetomidine, and ketamine.

Finally, once the imaging studies are completed, the patient should be monitored in a recovery unit until they have sufficiently recovered to their pre-procedure baseline level of activity. These units should have nursing coverage which allows close monitoring of cardiac, respiratory, and neurologic function, and the patient should demonstrate autonomous regulation of oxygenation, ventilation, circulation, and temperature regulation prior to discharge. As post-anesthesia nausea and vomiting is a common side effect, patients should be assessed for adequate volume status and ability to tolerate oral intake prior to discharge. In patients with comorbid disease, prolonged observation in an inpatient setting may be warranted.

## Special considerations for pre-term and term neonates undergoing anesthesia/sedation for imaging

Neonates and infants deserve special consideration regarding the use of pharmacologic agents to facilitate imaging acquisition. This is especially true for preterm neonates, whose brain development is immature at birth and continues ex utero, characterized by shifting populations of neuronal receptors (NMDA, GABA) distinct from older children and adults [41–46]. Many agents used to induce sedation and anesthesia act on NMDA and GABA receptors and have been associated with neuronal apoptosis in preclinical studies of young animals, leading to the previously mentioned FDA safety communication regarding sedation and anesthesia in children less than 3 years of age [47, 48]. Reassuringly, randomized controlled trial and longitudinal cohort data do not link brief anesthetic exposure to later neurodevelopmental delay [49–51].

As previously discussed, nonpharmacologic approaches, such as feed-and-swaddle and immobilization have been studied to limit movement during motion-sensitive studies (typically MRI). These succeed more than 90% of the time and are most successful in neonates and infants less than 3 months old [52–54]. Published protocols are available for reference [55]. This approach lessens the risk for adverse events and is therefore preferred when feasible. In some situations, particularly in ill or older infants, sedation or anesthesia is necessary to achieve diagnostic-quality images.

Risks associated with anesthesia and sedation in infants are similar to those of other pediatric patients, the most significant being cardiorespiratory depression due to hypoventilation, hypoxemia, and/or hypotension and hypothermia. Adverse events occur more in infants than older children, and preterm infants are at the highest risk regardless of corrected gestational age [56]. Respiratory complications such as apnea, oxygen desaturations, and airway obstruction are most frequently reported [56].

The increased risk for these complications likely relates to physiologic differences between infants and children. Preterm infants have decreased chemosensitivity to hypercarbia and an immature, altered response to hypoxia and apnea is common [56, 57]. Apneic pauses lead to atelectasis and a reduced functional residual capacity due to their highly compliant chest walls—this lower functional residual capacity yields faster oxygen desaturation [58]. As a result, all infants, and particularly those born premature, are at risk for hypoxic bradycardia [57, 59]. Additionally, infants and neonates are less able to respond to hemodynamic changes such as hypotension with augmentation of cardiac output compared to older children. Their ventricular compliance and thus ability to increase stroke volume is lower, so they rely on increasing heart rate [60]. In premature infants, side effects of commonly used sedating medications may be exacerbated. For example, propofol and midazolam frequently lead to hypotension, and fentanyl has been shown to have wide inter-individual variation in plasma concentration as well as half-life, though overall less effect on hemodynamics [61–64].

Thermoregulation is also an important consideration in infants where a high surface area to volume ratio increases risk for hypothermia. This can lead to downstream effects including bradycardia, altered response to adrenergic input, altered glucose metabolism, and decreased cerebral blood flow among others [65]. The use of a thermal mattress, swaddling blankets, and regular temperature monitoring is prudent to maintain normothermia.

Some critically ill neonatal populations deserve special attention when considering sedation and anesthesia selection. Infants with hypoxemic encephalopathy undergoing therapeutic hypothermia experience altered physiology including bradycardia and subsequent lower cardiac output, as well as decreased cerebral blood flow and metabolic rate, making careful hemodynamic monitoring especially important [65, 66]. Furthermore, changes in pharmacokinetics and pharmacodynamics lead to longer half-lives and lower rates of medication clearance [65, 67, 68]. The hypoxic injury itself may affect renal clearance and hepatic metabolism [67, 69]. These infants are at risk for drug accumulation and side effects, making dosing especially challenging. Overall, in these infants, non-urgent radiologic studies are avoided during hypothermia in order to maintain strict temperature control.

For infants on extracorporeal membrane oxygenation (ECMO), limited data exists to support optimal medication choice and dose. Critical illness itself can affect pharmacokinetics and pharmacodynamics and these complicating effects are intensified by adding ECMO. ECMO will increase volume of distribution and can sequester drugs, reducing plasma concentration [70, 71]. Travel to radiologic studies is generally avoided during ECMO, unless emergent, in which case sedation boluses alongside neuromuscular blockade can be utilized to prevent dislodgement of ECMO cannulas. Furthermore, most ECMO devices are not compatible with MRI machines, though there are currently MRI-compatible ECMO devices in development.

## Conclusion

It is important that radiologists consider this discussion in their evaluation of the necessity of imaging of the infant or neonate under sedation or anesthesia. There are often many ways to accomplish safe and effective sedation or anesthesia for imaging, and during this pursuit, one should also be considerate of the culture and resource base of the institution in which they practice. Some studies may be safely conducted by a sedation-trained provider, but for studies requiring longer times under anesthesia, more extensive pharmacologic or mechanical support, or in smaller, younger, or more critically ill infants and neonates, one should consider consultation with a fellowship-trained pediatric anesthesiologist. In addition, radiologists should be available for consultation with ordering providers to discuss the ideal imaging study and to help balance benefits of imaging with risks of obtaining imaging.

Additionally, safety considerations and practices in this population are similar to those for older children: infants should have intravenous access, continuous vital sign monitoring, a period of fasting prior to medication administration, emergency airway and resuscitation equipment available, and a clinician adept in airway management performing the sedation or anesthesia [14]. Infants and neonates are at a relatively higher likelihood of experiencing apnea, hypoxemia, hypotension, and hypothermia when subjected to anesthetic medications, and therefore general anesthesia, necessitating endotracheal intubation and mechanical or hand ventilation, is sometimes preferred over conscious/moderate sedation that is often better tolerated by older children. Furthermore, there are many known or suspected risks of anesthesia to the developing brain—it behooves physicians and providers to be cautious when considering the risks and benefits of sedation or anesthesia and imaging in this vulnerable population.

As radiology technology improves and expands, it is likely that the landscape of sedation and anesthesia for infant/neonatal imaging will change. The advent of certain technologies, such as contrast-enhanced ultrasound, may obviate some of the need for sedation or anesthesia by making fast bedside studies more commonly possible [72–76]. However, it is certain that there will be continued need for more intensive or invasive imaging in neonates and infants in the foreseeable future, and those involved in the request for or provision of these studies must understand the science and methods that make their performance possible.

## Supplementary Information

Below is the link to the electronic supplementary material.

Supplementary file1 (DOCX 24 KB)

## Data Availability

The authors confirm that the data supporting the conclusions contained within this review are available within the article, its supplementary materials, or within the referenced manuscripts.
