# Supplementary material for: Sedation and anesthesia for imaging of the infant and neonate—a brief review
Source: Pediatr Radiol. 2024 Jul 26;54(10):1579–88. doi: 10.1007/s00247-024-05995-5 (PMC11377638; doi:10.1007/s00247-024-05995-5)
Supplement: Supplementary file 1 — Supplementary file1 (DOCX 24 KB) [file 247_2024_5995_MOESM1_ESM.docx]

**Sedation and Anesthesia for Imaging of the Infant and Neonate – a Brief Review**

Supplementary Material

| ^Clear Liquids (Water, Juice, Jell-O, etc)^ | ^2 hours^ |
| --- | --- |
| ^Breastmilk^ | ^4 hours^ |
| ^Baby Formula^ | ^6 hours^ |
| ^Non-human Milk (Cow’s, Goat, etc)^ | ^6 hours^ |
| ^Light Meal (e.g. Toast, Cereal, etc)^ | ^6 hours^ |
| ^Heavier Meal (Meat, Deep Fried Foods, etc)^ | ^8 or more hours^ |

Supplementary Material 1: Adapted from ASA Fasting Recommendations [1].

| ASA Physical Status Classification | Definition | Adult Examples, Including but not Limited to: | Pediatric Examples, Including but not Limited to: |
| --- | --- | --- | --- |
| ASA 1 | A normal healthy patient | Healthy, active, minimal tobacco or alcohol use | Healthy, non-obese with no chronic medical problems |
| ASA 2 | A patient with mild systemic disease | - Mild systemic disease without noticeable functional impact - Active tobacco use - Active (social) alcohol use - Actively Pregnant - Mild to Moderate Obesity (e.g. BMI 30-40) - Controlled Diabetes or Hypertension - Mild, well-controlled respiratory disease (e.g Asthma, OSA) | - Asymptomatic/well controlled/corrected congenital or acquired cardiac disease - Well-controlled respiratory disease (e.g. Asthma, OSA), without recent exacerbations - Well controlled neurologic disease (e.g. epilepsy) - Non-insulin dependent diabetes - Mild-moderate obesity for age - Cancer (solid or liquid) in remission - Autism with mild limitations |
| ASA 3 | A patient with severe systemic disease | - Significant functional limitations - One or multiple moderate to severe diseases - Suboptimally controlled Diabetes or Hypertension - COPD - Morbid obesity (BMI ≥40) - Active hepatitis - Significant, routine alcohol use (e.g. dependence or abuse) - Implanted pacemaker - Moderately reduced ventricular ejection fraction - ESRD requiring routine renal replacement therapy (peritoneal or hemodialysis) - Past (>3 months) MI, CVA, TIA, or CAD/stents. | - Uncorrected stable congenital cardiac abnormality - Suboptimally controlled respiratory disease (Asthma, OSA) with recent or frequent exacerbations - Suboptimally controlled neurologic disease (e.g. epilepsy, Muscular Dystrophy, Hydrocephalus) - Insulin dependent diabetes - Morbid obesity - Active cancer - ESRD - Cystic Fibrosis - History of organ transplantation - Brain/spinal cord malformation - Premature infants <60 weeks post-conceptual age - Autism with severe limitations - Congenital Metabolic disease - Difficult airway - Long term parenteral nutrition - Age <6 weeks |
| ASA 4 | A patient with severe systemic disease that is a constant threat to life | - Recent (<3 months) MI, CVA, TIA or CAD/stents - Poorly controlled coronary artery disease or severe valvular dysfunction - Heart failure with severely reduced ejection fraction - Shock, sepsis, DIC, ARDS - ESRD not undergoing regularly scheduled dialysis | - Symptomatic congenital cardiac abnormality - Congestive heart failure - Active comorbidity related to  prematurity (Respiratory distress, ROP, growth failure, etc) - Acute hypoxic-ischemic encephalopathy - Shock, sepsis, DIC, Respiratory Disress - Implanted pacemaker or defibrillator - Ventilator dependence - Endocrinologic (Thyroid, Adrenal, Parathyroid, etc) disease - Severe trauma - Advanced cancer |
| ASA 5 | A moribund patient who is not expected to survive without the operation | - Ruptured AAA/TAA - massive trauma - Intracranial bleeding with mass effect - Multiorgan dysfunction/low output state | - Massive trauma - Intracranial hemorrhage with mass effect - ECMO - Respiratory failure/ARDS - Malignant hypertension Decompensated CHF - Hepatic encephalopathy - Multiorgan dysfunction |
| ASA 6 | A declared brain dead patient for organ donation |  |  |

Supplementary Material 2: ASA Physical Classification (Adapted from American Society of Anesthesiologists, Committee on Economics, 2014) [2].

| S | **SUCTION-**Size appropriate devices and cannisters on, functioning and readily accessible |
| --- | --- |
| O | **OXYGEN**- adequate supply (ideally pipeline and backup tank), and functioning flow meter, with appropriate delivery devices (nasal cannula, non-rebreather, BVM, etc) |
| A | **AIRWAY EQUIPMENT**- size appropriate devices (Bag-Valve-Mask, face mask, Naso/Oropharyngeal Airways, LMA, ETT, Laryngoscope, Stylet) with additional larger and smaller sizes readily available |
| P | **PHARMACY**- Medications necessary to achieve sedative hypnotic state, as well as treat potential complications (e.g. vasopressors, muscle relaxants, opioid and benzodiazepine antagonists, code medications) |
| M | **MONITORS**- ECG, NIBP, SpO2, EtCO2, Temperature monitoring readily available |
| E | **SPECIAL EQUIPMENT**- anything additional specific to the procedure that may be necessary |

Supplementary Material 3: AAP recommended equipment (Adapted from Coté & Wilson, 2019) [3].

1. (2017) Practice Guidelines for Preoperative Fasting and the Use of Pharmacologic Agents to Reduce the Risk of Pulmonary Aspiration: Application to Healthy Patients Undergoing Elective Procedures: An Updated Report by the American Society of Anesthesiologists Task Force on Preoperative Fasting and the Use of Pharmacologic Agents to Reduce the Risk of Pulmonary Aspiration*. Anesthesiology 126:376–393. https://doi.org/10.1097/ALN.0000000000001452

2. ​Statement on ASA Physical Status Classification System. https://www.asahq.org/standards-and-practice-parameters/statement-on-asa-physical-status-classification-system. Accessed 25 Sep 2023

3. Coté CJ, Wilson S, AMERICAN ACADEMY OF PEDIATRICS, AMERICAN ACADEMY OF PEDIATRIC DENTISTRY (2019) Guidelines for Monitoring and Management of Pediatric Patients Before, During, and After Sedation for Diagnostic and Therapeutic Procedures. Pediatrics 143:e20191000. https://doi.org/10.1542/peds.2019-1000
